# Supplementary material for: Ciprofloxacin-Loaded Zein/Hyaluronic Acid Nanoparticles for Ocular Mucosa Delivery
Source: Pharmaceutics. 2022 Jul 27;14(8):1557. doi: 10.3390/pharmaceutics14081557 (PMC9332751; doi:10.3390/pharmaceutics14081557)
Supplement: Supplementary file 1 [file pharmaceutics-14-01557-s001.zip › pharmaceutics-1820303-supplementary.pdf]

Ciprofloxacin-Loaded Zein/Hyaluronic Acid Nanoparticles for Ocular Mucosa Delivery

Telma A. Jacinto[1,#], Breno Oliveira[1,#], Sónia P. Miguel[1,2], Maximiano P. Ribeiro[1,2] and Paula Coutinho[1,2,*]

1 CPIRN-UDI/IPG, Centro de Potencial e Inovação em Recursos Naturais, Unidade de Investigação para o Desenvolvimento do Interior do Instituto Politécnico da Guarda, Avenida Dr. Francisco de Sá Carneiro, No. 50, 6300-559 Guarda, Portugal; telmajacinto@ipg.pt (T.A.J.); Brennofcb@hotmail.com (B.O.) spmiguel@ipg.pt (S.P.M.); mribeiro@ipg.pt (M.P.R.)

2 CICS-UBI, Centro de Investigação em Ciências da Saúde, Universidade da Beira Interior, Avenida Infante D. Henrique, 6200-506 Covilhã, Portugal;

* Correspondence: coutinho@ipg.pt.

# Authors contributed equally to this work.

Supplementary Information

3. Results and Discussion

3.1. TGA and DSC Analysis

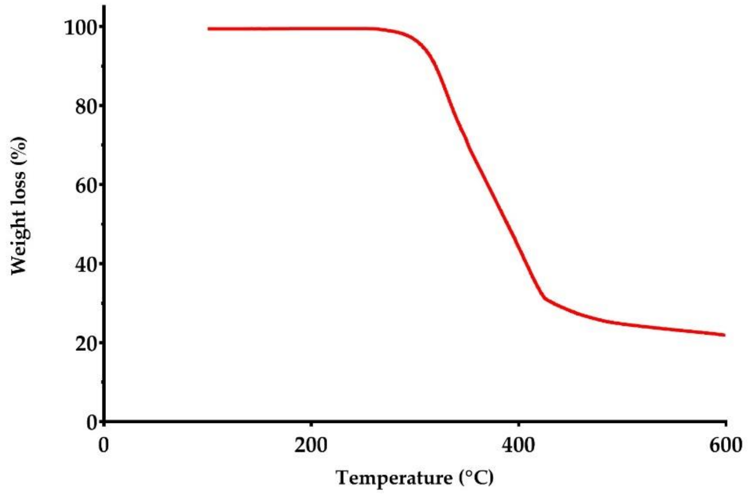

Figure S1. TGA analysis of ciprofloxacin.

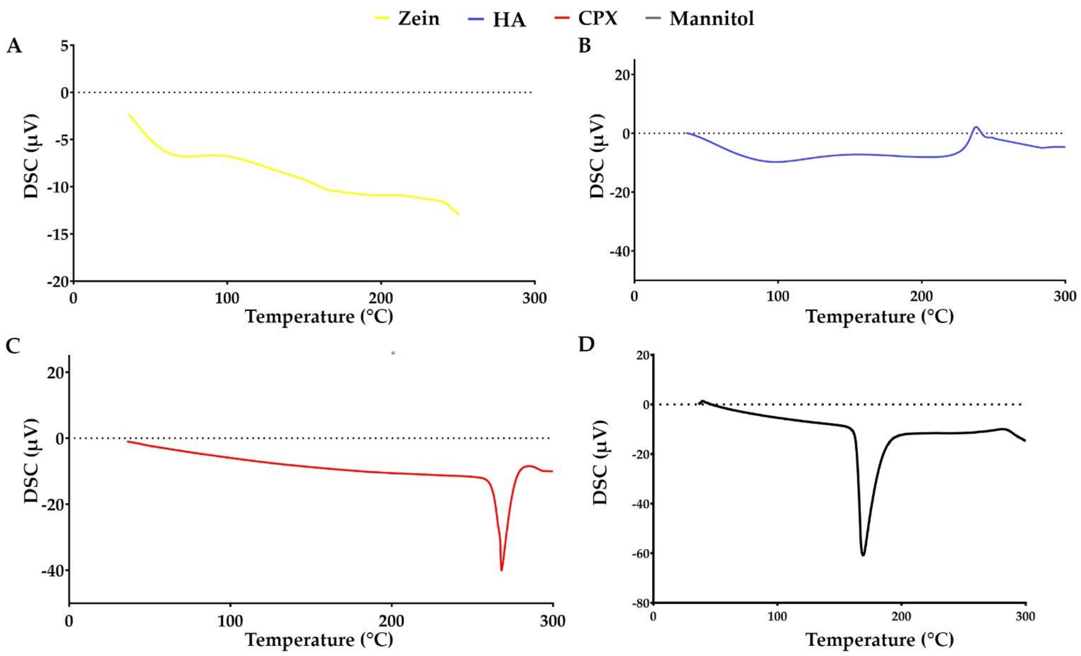

Figure S2. DSC thermograms of zein (A), hyaluronic acid (B), ciprofloxacin (C) and mannitol (D).
